# Comparison of Reverse Transcription (RT)-Quantitative PCR and RT-Droplet Digital PCR for Detection of Genomic and Subgenomic SARS-CoV-2 RNA

**DOI:** 10.1128/spectrum.04159-22

**Published:** 2023-03-21

**Authors:** Sara Morón-López, Eva Riveira-Muñoz, Victor Urrea, Lucia Gutiérrez-Chamorro, Carlos Ávila-Nieto, Marc Noguera-Julian, Jorge Carrillo, Oriol Mitjà, Lourdes Mateu, Marta Massanella, Ester Ballana, Javier Martinez-Picado

**Affiliations:** a IrsiCaixa AIDS Research Institute, Badalona, Spain; b CIBER de Enfermedades Infecciosas, Madrid, Spain; c Germans Trias i Pujol Research Institute (IGTP), Badalona, Spain; d Fight Infections Foundation, Badalona, Spain; e Hospital Universitari Germans Trias i Pujol, Badalona, Spain; f University of Vic-Central University of Catalonia (UVic-UCC), Vic, Spain; g Lihir Medical Centre, International SOS, Londolovit, Lihir Island, Papua New Guinea; h Autonomous University of Barcelona (UAB), Barcelona, Spain; i Catalan Institution for Research and Advanced Studies (ICREA), Barcelona, Spain; Fundacio irsiCaixa

**Keywords:** genomic SARS-CoV-2, subgenomic SARS-CoV-2, RT-qPCR, RT-ddPCR, viral persistence, long COVID, post-COVID-19 condition

## Abstract

Most individuals acutely infected with severe acute respiratory syndrome coronavirus 2 (SARS-CoV-2) exhibit mild symptoms. However, 10 to 20% of those infected develop long-term symptoms, referred to as post-coronavirus disease 2019 (COVID-19) condition (PCC). One hypothesis is that PCC might be exacerbated by viral persistence in tissue sanctuaries. Therefore, the accurate detection and quantification of SARS-CoV-2 are not only necessary for viral load monitoring but also crucial for detecting long-term viral persistence and determining whether viral replication is occurring in tissue reservoirs. In this study, the sensitivity and robustness of reverse transcription (RT)-droplet digital PCR (ddPCR) and RT-quantitative PCR (qPCR) techniques have been compared for the detection and quantification of SARS-CoV-2 genomic and subgenomic RNAs from oropharyngeal swabs taken from confirmed SARS-CoV-2-positive, SARS-CoV-2-exposed, and nonexposed individuals as well as from samples from mice infected with SARS-CoV-2. Our data demonstrated that both techniques presented equivalent results in the mid- and high-viral-load ranges. Additionally, RT-ddPCR was more sensitive than RT-qPCR in the low-viral-load range, allowing the accurate detection of positive results in individuals exposed to the virus. Overall, these data suggest that RT-ddPCR might be an alternative to RT-qPCR for detecting low viral loads in samples and for assessing viral persistence in samples from individuals with PCC.

**IMPORTANCE** We developed one-step reverse transcription (RT)-droplet digital PCR (ddPCR) protocols to detect SARS-CoV-2 RNA and compared them to the gold-standard RT-quantitative PCR (RT-qPCR) method. RT-ddPCR was more sensitive than RT-qPCR in the low-viral-load range, while both techniques were equivalent in the mid- and high-viral-load ranges. Overall, these results suggest that RT-ddPCR might be a viable alternative to RT-qPCR when it comes to detecting low viral loads in samples, which is a highly relevant issue for determining viral persistence in as-yet-unknown tissue reservoirs in individuals suffering from post-COVID conditions or long COVID.

## INTRODUCTION

Severe acute respiratory syndrome coronavirus 2 (SARS-CoV-2) typically causes mild symptoms in most acutely infected individuals (1). However, 10 to 20% of infected people experience long-term symptoms, referred to as post-coronavirus disease 2019 (COVID-19) condition (PCC) (2, 3). Although the etiology of PCC is unknown, it could be related to the persistence of viral antigens or RNA in tissue sanctuaries (4, 5). Therefore, the accurate detection and quantification of SARS-CoV-2 are crucial not only for monitoring viral loads (VLs) but also for detecting long-term viral persistence and differentiating whether there is active viral replication or only some residual viral antigens or RNA.

Reverse transcription (RT)-quantitative real-time PCR (qPCR) is considered the gold-standard method for the diagnosis of SARS-CoV-2 infection and is routinely used for epidemiological screening of individuals with suspected COVID-19. However, the sensitivity of RT-qPCR may be insufficient to detect viral persistence in samples with very low SARS-CoV-2 loads (6–8).

Furthermore, qPCR has been shown to produce variable data between laboratories (9, 10). In contrast, as droplet digital PCR (ddPCR) is based on absolute measurements, its intra- and interlaboratory variabilities are negligible. Therefore, RT-ddPCR enables the “absolute” quantification of the target to be amplified, is less dependent on PCR efficiency, allows the detection of sequence mismatches, and may be more sensitive and precise than RT-qPCR (11–13). Thus, the high tolerance to inhibitors, reproducibility, accuracy, and sensitivity of ddPCR compared to qPCR make the former a better technology when trying to quantify residual or low levels of the target.

Subgenomic SARS-CoV-2 RNA (sgRNA) has been reported as a marker of active viral replication (14) and may be a reliable indicator of viral persistence. However, RT-qPCR cannot quantify sgRNA because of the lack of a proper standard. Therefore, more sensitive and robust detection methods are needed, especially for samples with low and residual viral loads, to accurately diagnose and treat of SARS-CoV-2 and to mitigate the limitations of RT-qPCR. In this context, after limiting dilution and endpoint PCR, RT-ddPCR provides absolute quantification through Poisson statistics, which represents a more accurate measurement than RT-qPCR (6–8, 11–13).

In this study, we compared SARS-CoV-2 genomic RNA (gRNA) and subgenomic RNA detection from oropharyngeal swab samples from confirmed SARS-CoV-2-infected, SARS-CoV-2-exposed, and nonexposed individuals and samples from mice infected with SARS-CoV-2 using both RT-qPCR and RT-ddPCR. Our findings indicate a correlation between SARS-CoV-2 detection and quantification using RT-qPCR and RT-ddPCR and a higher sensitivity of RT-ddPCR for the detection of cases of SARS-CoV-2 infection in exposed individuals with negative RT-qPCR results.

## RESULTS

### RT-ddPCR SARS-CoV-2 quantification was similar to that of RT-qPCR.

**FIG 1:**
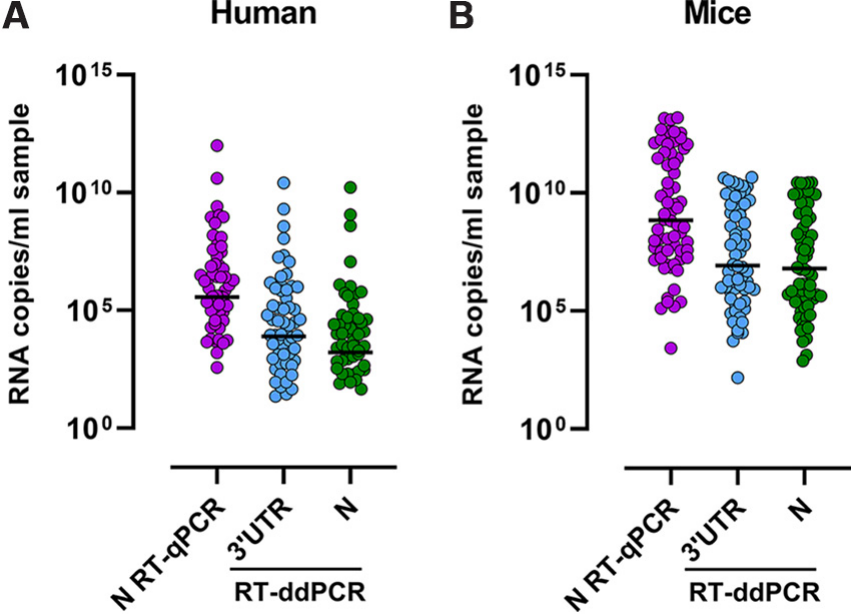
Levels of RT-qPCR and RT-ddPCR quantifications. SARS-CoV-2 RNA copies per milliliter of sample were quantified using RT-qPCR (N) and RT-ddPCR (3′ UTR and N) in human (A) and mouse (B) samples.

**FIG 2:**
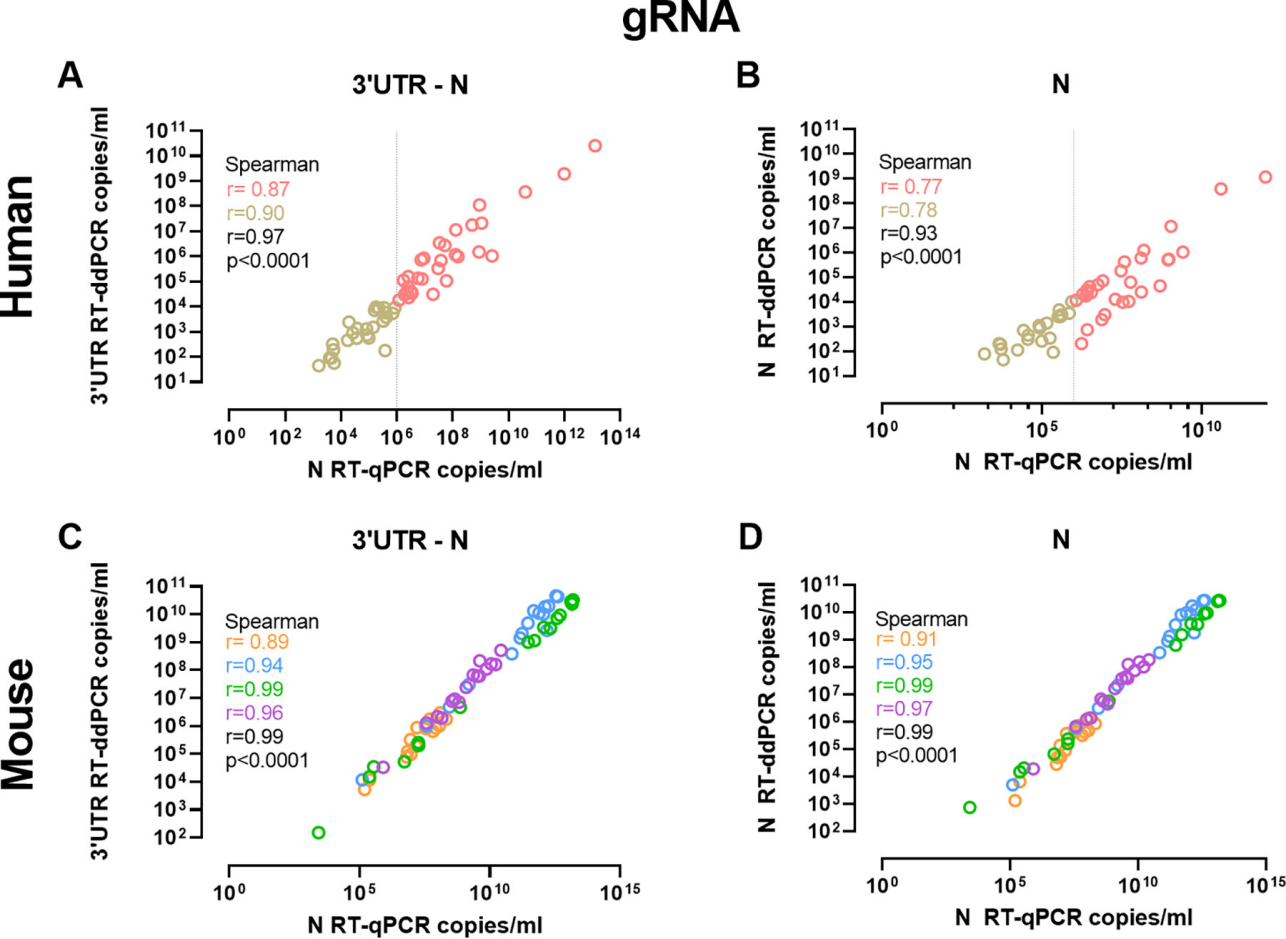
Correlation between RT-qPCR and RT-ddPCR quantifications. Comparisons were performed between gRNA (N) quantified by RT-qPCR and 3′-UTR and N quantifications by RT-ddPCR in human samples (A and B) and mouse samples (C and D). Spearman correlations are presented, and *r* and *P* values are specified in each plot. Colors show *C_T_* stratifications in human samples (brown, *C_T_* value of ≤30; pink, *C_T_* value of >30) and mouse samples from different tissues (orange, oropharyngeal swab; blue, lung; green, brain; purple, nasal turbinate).

**FIG 3:**
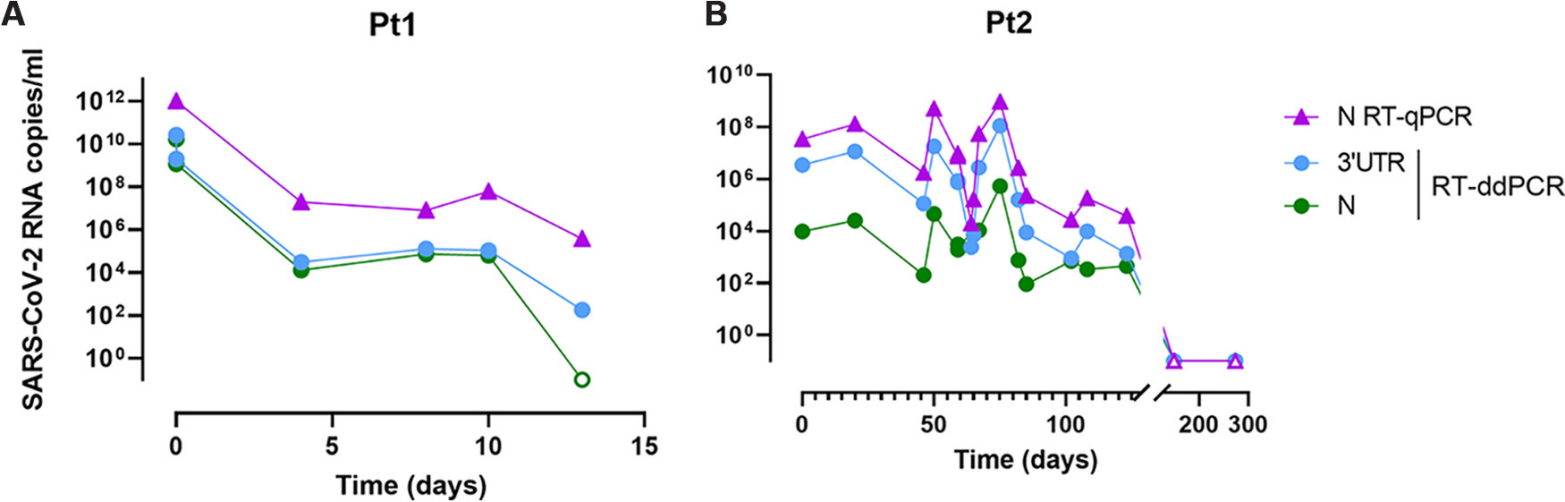
Longitudinal follow-up of SARS-CoV-2 loads in nasopharyngeal swabs. Quantification of the N region was performed by RT-qPCR, and quantifications of the N region and the 3′ UTR were performed by RT-ddPCR in participant 1 (Pt1) (A) and participant 2 (B). Open symbols represent values below the limit of detection.

In order to assess whether RT-ddPCR SARS-CoV-2 detection was equivalent to that of the gold-standard RT-qPCR, we quantified and compared the levels of SARS-CoV-2 RNA (genomic and subgenomic) in 98 nasopharyngeal swabs from confirmed SARS-CoV-2-infected individuals (*n* = 68), SARS-CoV-2-exposed but SARS-CoV-2-negative subjects (*n* = 10), and non-SARS-CoV-2-exposed individuals (*n* = 8) and in 64 samples obtained from SARS-CoV-2-infected K18-hACE2 mice by both techniques. When comparing the levels of SARS-CoV-2 RNA, RT-qPCR values were higher than RT-ddPCR values in samples from both humans and mice (Fig. 1A to B). Nonetheless, RT-ddPCR results for genomic SARS-CoV-2 RNA were strongly correlated with RT-qPCR data irrespective of the origin of the sample (human or mouse) or stratification by the threshold cycle (*C_T_*) or mouse tissue (*P* < 0.0001; rho value of >0.77) (Fig. 2A to D; see also Fig. S1 in the supplemental material). When we analyzed subgenomic SARS-CoV-2 RNA results, RT-ddPCR detection correlated with RT-qPCR detection in human nasopharyngeal swabs (*P* < 0.0001; rho value of less than −0.76) (Fig. S2A and B) and with both RT-ddPCR and RT-qPCR genomic SARS-CoV-2 RNA quantifications in mice (*P* < 0.0001; rho value of >0.92) (Fig. S2C to H). We observed similar results when using regression models (Fig. S1) and when comparing specific quantifications of gRNA and sgRNA (Fig. S2). Furthermore, we compared the detection of SARS-CoV-2 RNA in longitudinal nasopharyngeal swabs from two individuals (Fig. 3A to B). We observed that the quantification and dynamics using RT-ddPCR were similar to those determined using RT-qPCR. Altogether, these results suggest that the detection of SARS-CoV-2 RNA using RT-ddPCR is equivalent to that of the gold-standard RT-qPCR and allows the absolute quantification of subgenomic SARS-CoV-2 RNA.

### RT-ddPCR showed higher sensitivity in exposed individuals and allowed the detection of sequence variability.

**FIG 4:**
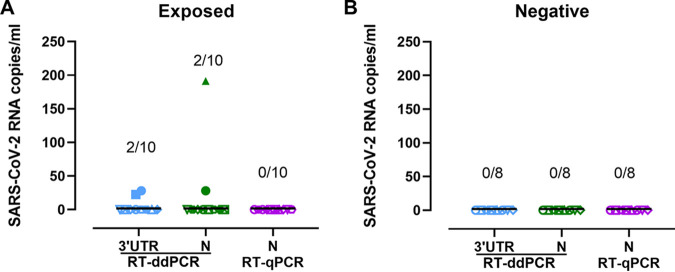
Quantification of SARS-CoV-2 loads in nasopharyngeal swabs from exposed and nonexposed individuals. Shown are quantifications of SARS-CoV-2 RNA targeting the 3′ UTR and the N region by RT-ddPCR and the N region by RT-qPCR in exposed (A) and nonexposed/negative (B) individuals. Symbols represent samples from different individuals.

To determine whether RT-ddPCR is more sensitive than RT-qPCR, we compared SARS-CoV-2 detection in nasopharyngeal swabs from confirmed SARS-CoV-2-exposed (*n* = 10) and nonexposed (*n* = 8) individuals. RT-qPCR detections were all negative for both groups, while RT-ddPCR quantifications were positive for three samples from the exposed individuals (Fig. 4A), with one being detected with both sets of primers/probes (N gene and 3′ untranslated region [UTR]). These data suggest that RT-ddPCR might be more sensitive than RT-qPCR in samples with low viral loads. Furthermore, these data confirmed that both RT-qPCR and RT-ddPCR are robust techniques, as no false-positive results were detected in the negative group (Fig. 4B).

Additionally, using RT-ddPCR, we were able to observe possible sequence mismatches in nasopharyngeal swabs from individuals from the confirmed SARS-CoV-2-positive cohort (Fig. S3) using either the 3′-UTR primer/probe set or the N set. Specifically, in sample 2, as we were able to sequence the virus, we aligned the primer and probe sequences to the viral sequence, and we observed a mismatch at the 3′ end of the N forward primer (5′-CATCACGTAGTCGCAACAG-3′), in which G was replaced with C (sequence, 5′-CATCACGTAGTCGCAACAC-3′) (see the supplemental material). Thus, RT-ddPCR allows not only the determination of the absolute quantification of a sample but also the detection of sequence mismatches affecting PCR performance and, more importantly, enables the assessment of viral evolution in longitudinal samples from the same individual.

## DISCUSSION

Since the SARS-CoV-2 outbreak began, a wide range of diagnostic tools have been developed to study this virus, and the current gold standards are PCR-based assays. However, these methods are semiquantitative, do not account for variation in gene copy numbers due to subgenomic transcription, and are usually limited to the detection of one or two regions of the genome (15). In the present study, we compared the accuracy, sensitivity, and robustness of the gold-standard RT-qPCR to those of RT-ddPCR for the detection of SARS-CoV-2 genomic and subgenomic RNAs. We analyzed oropharyngeal swab samples from confirmed SARS-CoV-2-positive, SARS-CoV-2-exposed, and nonexposed individuals as well as samples from mice with SARS-CoV-2 infection. Our results indicate that RT-ddPCR quantification is equivalent to RT-qPCR quantification for both the high- and low-viral-load ranges. Additionally, ddPCR displayed higher sensitivity, which allowed the detection of very low levels of SARS-CoV-2 RNA in exposed individuals without detecting false positives in the nonexposed controls. We used one-step RT-ddPCR with multiplexed primer/probe sets targeting N and the 3′ UTR, which detected both genomic and subgenomic SARS-CoV-2 RNAs (15), and with single primer/probe sets targeting either genomic or subgenomic RNA for the N gene (16) and the gold-standard RT-qPCR with primer/probe sets targeting N for genomic RNA and E for subgenomic RNA (17).

Our objective was to compare the performance of RT-ddPCR with that of RT-qPCR for several reasons: first, ddPCR allows absolute quantification, without the need for an external calibrator; second, RT-ddPCR tends to tolerate sequence mismatches in primer/probe sequences better than RT-qPCR and also allows the visualization of sequence mismatches by comparison of the fluorescence levels of the sequences amplified; and third, it may be more precise at low copy numbers while providing sensitivity and reproducibility similar to those of qPCR (18, 19).

When comparing RT-ddPCR and RT-qPCR quantifications, we observed that RT-qPCR values were higher than RT-ddPCR values. Considering that ddPCR provides absolute quantification of the amplified target, this lower value may reflect inaccurate extrapolation when quantifying by a standard curve or differences in PCR efficiencies (20, 21). However, Telwatte et al. (15) compared the performances of the primer/probe sets used in this work for RT-ddPCR with those used for RT-qPCR and reported similar sensitivities.

The added value of multiplexing assays that target different RNA SARS-CoV-2 regions is supported by studies that demonstrate a decreased sensitivity or loss of detection of published primer/probe sets due to mutations affecting primer annealing. Peñarrubia et al. (22) observed that more than 34% of SARS-CoV-2 genomes had a single mutation that may have affected annealing in assays from the World Health Organization, the Centers for Disease Control and Prevention (CDC), the National Microbiology Data Center, and Hong Kong University. Furthermore, Vogels et al. (23) found single nucleotide mismatches in 0.2 and 0.4% of SARS-CoV-2 sequences compared to the CDC-N1 probe and reverse primer, respectively, and 0.4% of viral sequences compared to Charité’s E Sarbeco R primer.

PCR-based assays commonly used in clinical settings are quite sensitive for the detection of SARS-CoV-2 infection during the first 1 to 3 weeks but often become negative afterward (24–26), suggesting that the virus has been cleared a few weeks after infection onset. Nevertheless, some people, regardless of whether they are immunocompromised or not, have persistent infection or persistent shedding of viral RNA in the long term (27–32). Viral persistence is one of the potential pathophysiological mechanisms contributing to PCC (33, 34), but the quantification of viral genomes in samples from individuals with PCC remains limited. Thus, a multiplex assay covering several regions that is capable of accurately detecting both genomic and subgenomic RNAs might be useful for increasing the sensitivity and detection of low-abundance SARS-CoV-2, while highly sensitive PCR-based methods have proven essential for detecting SARS-CoV-2 infection in individuals in the acute phase of the infection. Importantly, quantitative assays capable of detecting extremely low copy numbers of SARS-CoV-2 will be particularly useful for demonstrating viral persistence in people who have PCC and understanding long-term disease progression.

The limitations of this study should be acknowledged. First, the primer/probe sets used for ddPCR differ from the ones used for qPCR. Therefore, some inaccuracies in positivity might be due to sequence mismatches and not to assay/method performance. Second, the multiplexed assays of ddPCR quantify both genomic and subgenomic SARS-CoV-2 RNAs in order to increase sensitivity; however, these assays cannot differentiate whether the sequences detected come from a whole viral genome or small sequences of noninfectious viral RNA. Third, by multiplexing assays, there might be some amplification inhibition due to competition among primer and probe amplifications for the different assays, albeit we compared singleplexing and multiplexing of the 3′-UTR and N assays, and we did not observe differences in quantifications (data not shown). Fourth, we should have also compared the performance of our multiplexed RT-ddPCR assay with that of a commercial multiplexed RT-qPCR assay; however, we could not do that due to sample availability.

Taken together, these results suggest that the use of RT-ddPCR might be a viable alternative to RT-qPCR when it comes to detecting low viral loads in samples, which is a highly relevant issue for determining viral persistence in as-yet-unknown tissue reservoirs in individuals suffering from PCC.

## MATERIALS AND METHODS

### Human samples.

Nasopharyngeal swabs used in this study were obtained from patients with confirmed SARS-CoV-2 infection (*n* = 68; median number of days from symptom onset, 12 [interquartile range {IQR}, 7 to 54 days]), PCR-negative SARS-CoV-2-exposed individuals (*n* = 10; median number of days since exposure, 21 [IQR, 20 to 23 days]), or PCR-negative non-SARS-CoV-2-exposed individuals (*n* = 8) from the BCN PEP CoV-2 study (ClinicalTrials.gov identifier NCT04304053) and the KING cohort extension.

The BCN PEP CoV-2 study and the KING cohort extension (PI-20-217) were approved by the Ethics Committee Boards of the Hospital Universitari Germans Trias i Pujol. All participants provided written informed consent.

Samples were selected in order to cover *C_T_* ranges equivalent to undetermined, low, medium, and high viral loads quantified by the gold-standard RT-qPCR (see Table S1 in the supplemental material).

### Mouse samples.

All animal procedures were performed under the approval of the Committee on the Ethics of Animal Experimentation of the IGTP and the authorization of the Generalitat de Catalunya (code 10965).

Oropharyngeal swab and tissue samples from the lung, brain, and nasal turbinates were collected from 16 SARS-CoV-2-infected K18-hACE2 mice (50% male/50% female, 7 to 9 weeks old; Jackson Laboratory) for viral load (VL) determination. Mice were challenged with 1,000 50% tissue culture infective doses (TCID_50_) of the SARS-CoV-2 D614G isolate, and tissue samples were collected on day 2, 4, or 7 after challenge or according to the humane endpoints defined in the supervision protocol (weight loss of >20%, drastic reduction in mobility, or significant reduction in the response to stimuli). Tissue samples were collected in 1.5-mL tubes containing Dulbecco’s modified Eagle’s medium (DMEM) supplemented with penicillin (100 U/mL) and streptomycin (100 μg/mL). Next, tissues were homogenized twice at 25 Hz for 30 s using a Tissue Lyser II instrument and a 1.5-mm Tungsten bead (Qiagen). After that, samples were centrifuged for 2 min at 2,000 × *g*, and the supernatants were collected and stored at −80°C until use.

### RNA extraction.

RNA extraction from frozen nasopharyngeal swabs and processed mouse tissue samples was performed by using a custom Maxwell HT viral TNA (total nucleic acid) kit (Promega), optimized for a KingFisher instrument (Thermo Fisher), according to the manufacturer’s instructions.

### SARS-CoV-2 RT-qPCR detection and viral load quantification.

PCR amplification was based on the 2019 novel coronavirus (2019-nCoV) real-time RT-PCR diagnostic panel guidelines and protocol developed by the U.S. Centers for Disease Control and Prevention (Fig. S4). Briefly, a 20-μL PCR mixture was set up, containing 5 μL of RNA, 1.5 μL of N2 primers and probe (2019-nCoV CDC emergency use authorization [EUA] kit, catalog number 10006770; Integrated DNA Technologies), and 10 μL of GoTaq one-step RT-qPCR mix (Promega). Thermal cycling was performed at 50°C for 15 min for reverse transcription, followed by 95°C for 2 min and then 45 cycles of 95°C for 3 s and 55°C for 30 s, in the Applied Biosystems 7500 or QuantStudio5 real-time PCR instrument (Thermo Fisher). For absolute quantification, a standard curve was built using 1/5 serial dilutions of a SARS-CoV-2 plasmid (200 copies/μL) (2019-nCoV_N_Positive Control, catalog number 10006625; Integrated DNA Technologies) and run in parallel for all PCR determinations. The viral load in each sample was determined in triplicate, and the mean viral load (in copies per milliliter) was extrapolated from the standard curve and corrected by the corresponding dilution factor. Human glyceraldehyde-3-phosphate dehydrogenase (GAPDH) gene amplification was performed in duplicate for each sample as an amplification control.

Testing for subgenomic RNA was performed using a leader-specific primer as well as primers and probes targeting sequences downstream of the start codons of the E gene, as previously described (17) (Fig. S4). The RT-PCR assay was performed using GoTaq one-step RT-qPCR (Promega) with 400 nM each primer and 100 nM probe. Thermal cycling was performed for 15 min at 50°C for reverse transcription, followed by 2 min at 95°C, 10 s at 95°C, 15 s at 56°C, and 30 s at 72°C. Human GAPDH gene amplification was performed in duplicate for each sample as an amplification control.

### SARS-CoV-2 RT-ddPCR quantification.

RT-ddPCR amplification was performed using the one-step RT-ddPCR advanced kit (Bio-Rad), according to the manufacturer’s instructions, using primer/probe sets targeting the N region and the 3′ UTR, which detect both genomic SARS-CoV-2 RNA (gRNA) and subgenomic SARS-CoV-2 RNA (sgRNA), and primer/probe sets targeting the N region, which detect either genomic or subgenomic SARS-CoV-2 RNA, designed previously by Telwatte et al. (15, 16) (Fig. S4). Briefly, for simultaneous gRNA and sgRNA detection, a 20-μL PCR mixture was set up, containing 4 μL of RNA, 1 μL of 3′-UTR primers and probes (20×), 1 μL of N_ORF9 primers and probes (20×), 1 μL of dithiothreitol (DTT) (300 nM), 5 μL of RT-ddPCR supermix, 2 μL of reverse transcriptase enzyme, and 6 μL of RNase/DNase-free water. Thermal cycling was performed at 50°C for 60 min for reverse transcription followed by 95°C for 10 min, 45 cycles of 95°C for 30 s and 54°C for 1 min, and a final step at 98°C for 10 min in the C1000 touch thermal cycler (Bio-Rad). For separate gRNA and sgRNA detection, a 20-μL PCR mixture was set up, containing 4 μL of RNA, 1 μL of gN primers and probes (20×) or sgN primers and probes (20×), 1 μL of DTT (300 nM), 5 μL of RT-ddPCR supermix, 2 μL of reverse transcriptase enzyme, and 6 μL of RNase/DNase-free water. After RT-ddPCR, the plate was immediately analyzed in the QX100 plate reader (Bio-Rad). RT-ddPCRs for each sample were performed in duplicate, and direct absolute quantification was performed using QuantaSoft 1.6.6.0320 software by merging both wells and correcting by the corresponding dilution factor. Positive (supernatants from *in vitro* infections with SARS-CoV-2) and negative (samples from SARS-CoV-2-uninfected individuals, no RT, and water) controls were included in each plate.

### Statistical analysis.

Spearman correlation analyses were performed using GraphPad Prism (v.9.3.0), while regression models for censored data using maximum likelihood estimation were performed using R.
