# Correction for Morón-López et al., “Comparison of Reverse Transcription (RT)-Quantitative PCR and RT-Droplet Digital PCR for Detection of Genomic and Subgenomic SARS-CoV-2 RNA”

**DOI:** 10.1128/spectrum.02269-24

**Published:** 2024-10-30

**Authors:** Sara Morón-López, Eva Riveira-Muñoz, Víctor Urrea, Lucia Gutiérrez-Chamorro, Carlos Ávila-Nieto, Marc Noguera-Julian, Jorge Carrillo, Oriol Mitjà, Lourdes Mateu, Marta Massanella, Ester Ballana, Javier Martinez-Picado

## AUTHOR CORRECTION

Volume 11, no. 2, e04159-22, 2023, https://doi.org/10.1128/spectrum.04159-22. Page 7, Acknowledgments: The following should be added at the end: “This work was funded by the European Union. Views and opinions expressed are, however, those of the author(s) only and do not necessarily reflect those of the European Union or the granting authority European Union’s Horizon Europe research and innovation program. Neither the European Union nor the granting authority can be held responsible for them. The PCR-4-ALL project has received funding under the Horizon Europe research and innovation program (grant agreement no. 101095606).”

